# Erysipelothrix rhusiopathiae: Blood, Bones, and the Beating Heart

**DOI:** 10.7759/cureus.81380

**Published:** 2025-03-28

**Authors:** Daniel Sherlock, Hannah Runnoe, Noor Alkhawam, Viktoriya Bikeyeva, Danny Cho, Tom Waller, Adib Chaus

**Affiliations:** 1 Internal Medicine, Advocate Lutheran General Hospital, Park Ridge, USA; 2 Internal Medicine, Advocate Christ Medical Center, Oak Lawn, USA; 3 Infectious Diseases, Advocate Lutheran General Hospital, Park Ridge, USA; 4 Cardiology, Advocate Lutheran General Hospital, Park Ridge, USA

**Keywords:** bioprosthetic aortic valve endocarditis, endocarditis, erysipelothrix rhusiopathiae, gram positive bacteremia, osteo-myelitis

## Abstract

*Erysipelothrix rhusiopathiae*, a Gram-positive bacillus, infrequently causes human infections. While localized cutaneous infections are most common, they can also lead to septicemia and/or endocarditis. In this case report, we discuss a patient initially presenting with back pain, confusion, and forgetfulness worsening over the month preceding admission. She was found to have a complex *E. rhusiopathiae* infection involving bacteremia complicated by bioprosthetic aortic valve endocarditis, native mitral valve endocarditis, lumbar discitis and osteomyelitis, and an abscess. The patient was noted to have a penicillin allergy, so ceftriaxone was chosen for appropriate coverage. The patient was not deemed a candidate for surgical or procedural interventions, so treatment with a six-week course of intravenous ceftriaxone was initiated, and she was noted to have resolved her infection with no further complications. This case not only adds to the sparse data on E. rhusiopathiae infections involving endocarditis and osteomyelitis but also prompts reconsideration of management strategies in the aging population with multiple comorbidities and implanted devices.

## Introduction

*Erysipelothrix rhusiopathiae* is a Gram-positive, rod-shaped bacterium known for its zoonotic potential. Although it rarely causes human infections, it is most commonly associated with cutaneous infections known with both local (erysipeloid) generalized cutaneous forms [1]. Nonetheless, cases of bacteremia and endocarditis have been reported [2]. This pathogen is typically found in domestic swine and various other animals such as fish and birds. Occupational exposures such as direct handling of fish or meat, gardening, farming, or cooking pose potential risks for infection. The organism is commonly found in decomposing organic matter and is remarkably durable, able to survive in soil for several weeks.

Bacteremia, the presence of bacteria in the blood, can lead to infectious seeding of various organs in the body including the heart, and has been associated with significant morbidity and mortality. Endocarditis is characterized by the formation of vegetation on heart valves (both native and prosthetic), which can lead to valvular destruction, extension of infection to the paravalvular space, and heart failure. Extracardiac complications include systemic embolization which can affect various organs including the brain, kidney, spleen, lungs, and vasculature leading to strokes, abscess formation, and other serious complications. Endocarditis from *E. rhusiopathiae* appears to involve mainly native aortic and mitral valves, with a male predominance [3]. *E. rhusiopathiae* can be difficult to diagnose by traditional culture methods due to its slow growth and small colony size. Treatment options for *E. rhusiopathiae* include penicillins, cephalosporins, erythromycin, and clindamycin, as most strains are susceptible to these antibiotics [2]. Notably, *E. rhusiopathiae* is resistant to vancomycin. Understanding the sources and risks associated with this pathogen can aid in the prompt recognition and management of these infections.

In this report, we present the case of an 86-year-old who was found to have *E. rhusiopathiae* bacteremia complicated by bioprosthetic aortic valve endocarditis, native mitral valve endocarditis, lumbar discitis and osteomyelitis, and an abscess. This case highlights the need for further research on *E. rhusiopathiae* infections, particularly in the context of endocarditis and osteomyelitis, and emphasizes the importance of tailored management strategies for aging patients with multiple comorbidities and implanted devices. 

## Case presentation

We present an 86-year-old female with a past medical history significant for coronary artery disease (CAD), status post coronary artery bypass grafting (CABG) 17 years prior; aortic valve stenosis, status post transcutaneous aortic valve implantation (TAVI); complete heart block, status post-Micra pacemaker placement (about one year prior to presentation); moderate mitral valve stenosis; diastolic heart failure; hypertension; and hyperlipidemia, who presented to the hospital with back pain, confusion, and urinary and fecal incontinence. The patient's husband was unable to provide much detail on her baseline mental status but did mention that she had become increasingly confused and had experienced recurrent falls leading up to this admission. Prior to her TAVI, she had been noted to have experienced shortness of breath with exertion, but she had also been unable to exert herself much due to osteoarthritis of her knees and significant lower extremity lymphedema.

One month prior to the presentation, the patient visited her primary care physician (PCP) due to severe left-sided back pain and weakness that had persisted for four days. The pain was particularly intense, radiating down her left leg, and had impaired her ability to walk at home. The night before her appointment, the patient experienced a suspected mechanical fall, though she denied hitting her head or back during the incident. Upon examination and evaluation, the PCP ordered X-rays of her spine, which revealed a compression deformity of the thoracic spine and multilevel arthritis of the lumbar spine. In response to these findings, the PCP recommended thoracic and lumbar magnetic resonance imaging (MRI) as well as home health physical therapy. However, the scheduling and approval process for the MRI were complicated by the patient’s Micra placement. 

**Figure 1:**
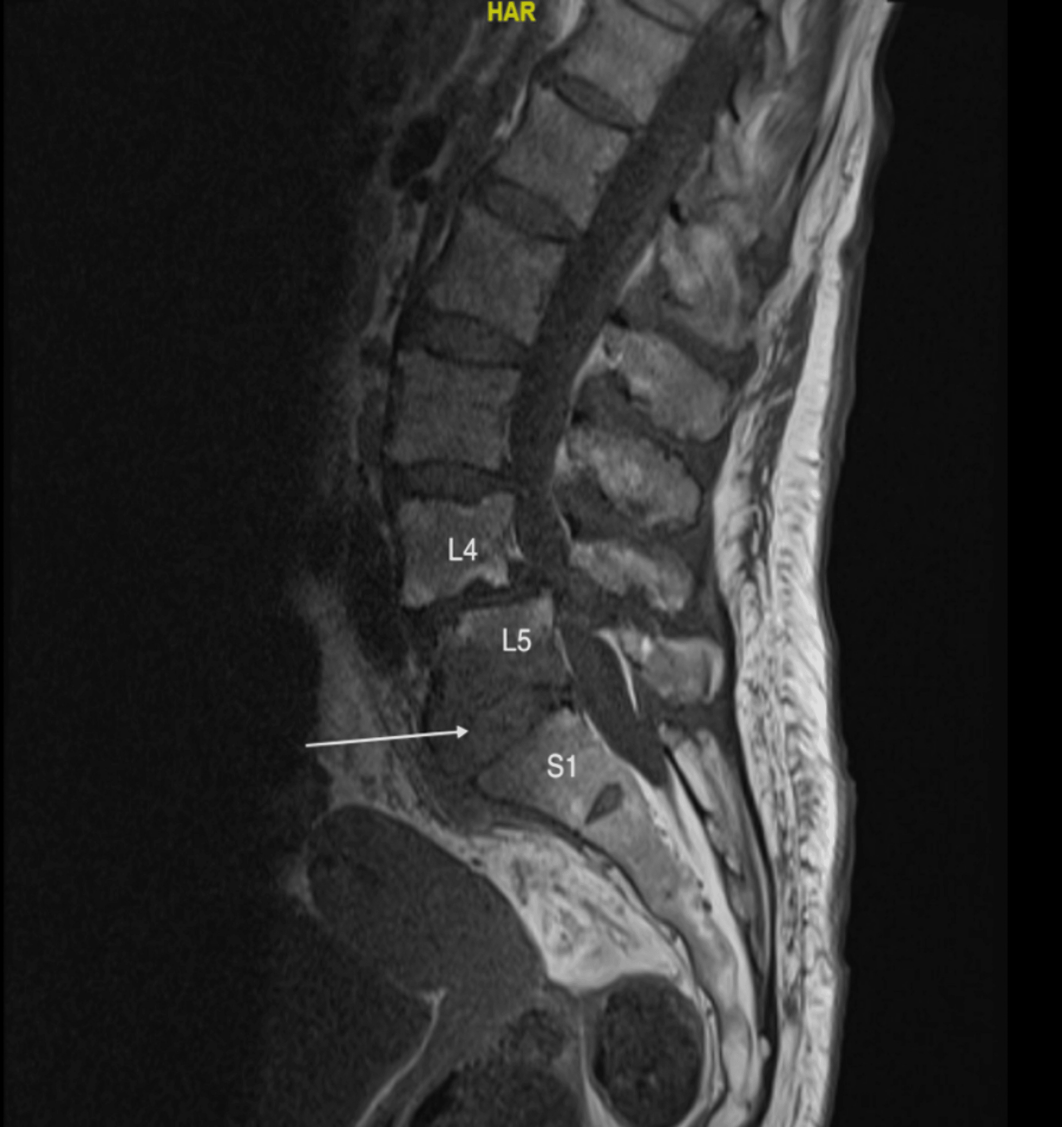
An MRI of the lumbar spine showed circumferential soft tissue fullness with irregularities and erosive findings of the superior and inferior endplates, consistent with discitis at the L5-S1 level (white arrow) MRI: magnetic resonance imaging

Approximately two weeks later, she had another office visit to her PCP for repeated falls, memory, and speech changes. She was found to have *E. coli* bacteriuria and was treated with a seven-day course of nitrofurantoin 100 mg twice a day. Five days later, she presented for her previously ordered thoracic and lumbar spine MRI, which showed findings suspicious for L5-S1 discitis (Figure 1).

In light of the MRI findings and ongoing symptoms, the patient was taken to the emergency department (ED) that day for further evaluation. The patient remained lethargic but would respond with grimacing to palpation of her back. The patient's family denied any recent sick contacts or travel history. The patient's husband stated that she had spent the past month at home and occasionally did some gardening, but she did not have any pets at home and did not cook. Initial notable laboratory findings are included in Table 1. 

**Table 1:** Notable laboratory findings on admission NTproBNP: N-terminal pro-b-type natriuretic peptide; ESR: erythrocyte sedimentation rate; CRP: C-reactive protein

Parameter	Patient value	Reference range
High-sensitivity troponin I	128 ng/L	<52 ng/L
NTproBNP	7898 pg/mL	≤450 pg/mL
ESR	63 mm/hr	0-20 mm/hr
CRP	4.7 mg/dL	≤1.0 mg/dL

**Figure 2:**
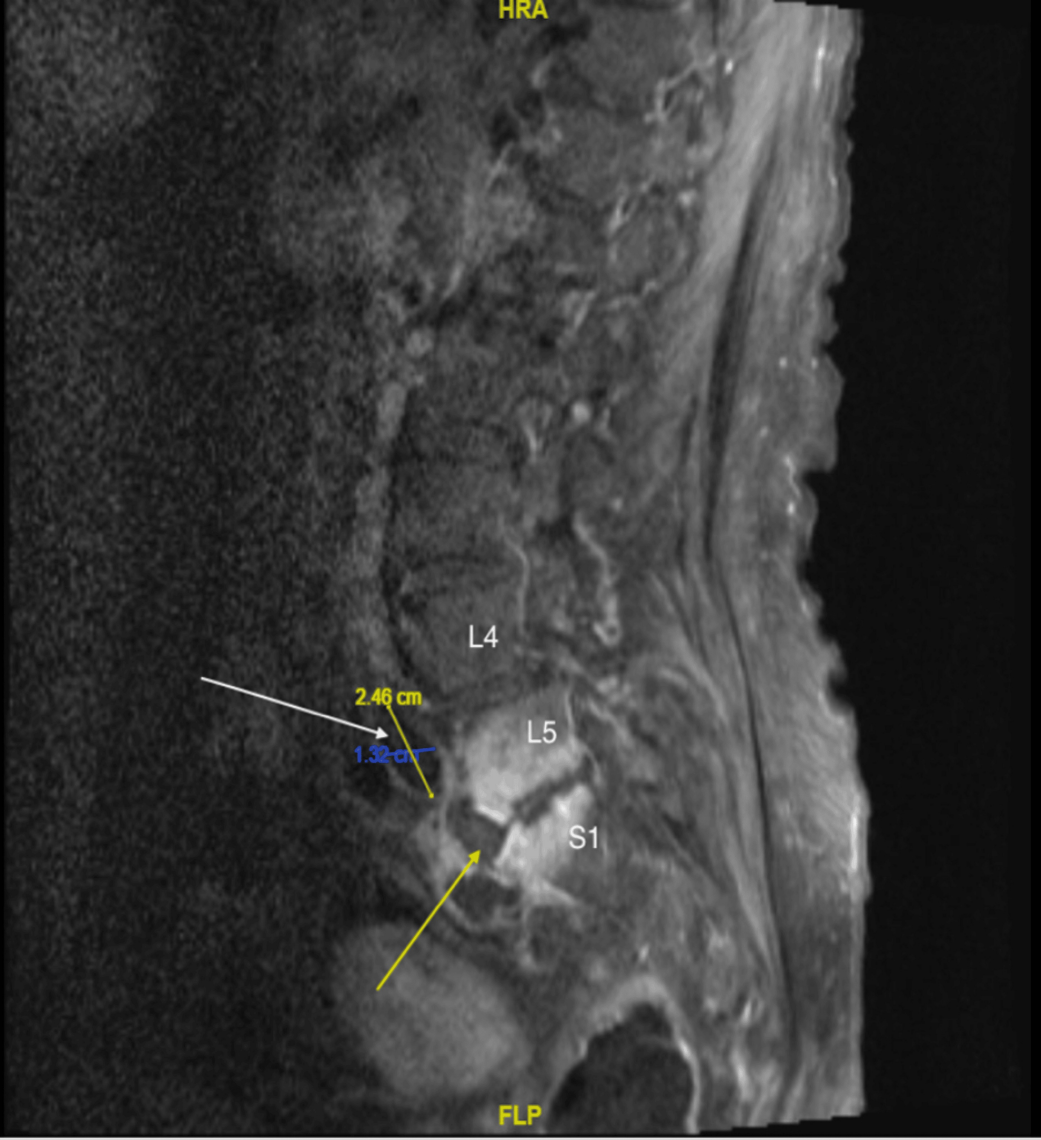
MRI of the lumbar spine showed a small abscess within the soft tissue (white arrow) along the ventral margin of the intervertebral disc and the anterior cortex of the sacrum, left of midline. Contrast enhancement of the endplates at L5-S1 is consistent with discitis (yellow arrow). The study was limited due to motion artifact MRI: magnetic resonance imaging

Initial electrocardiogram (EKG) revealed sinus tachycardia, right bundle branch block (RBBB), and left posterior fascicular block (LPFB). CT lumbar spine without contrast showed disc space widening, endplate erosions, and mild paraspinal soft tissue thickening suggestive of spondylodiscitis at the L5-S1 level. MRI revealed osteomyelitis/discitis at the L5-S1 level. There was a small abscess within the soft tissues, located ventral to the intervertebral disc space, left of midline, measuring 1.3 cm x 2.5 cm x 2.2 cm in anterior-posterior (AP), transverse, and craniocaudal dimensions, respectively (Figure 2).

Neurosurgery was consulted, and no surgical intervention was recommended. Her pre-TAVI cardiac catheterization did not show any obstructive CAD, but she developed new-onset atrial fibrillation. Anticoagulation was initially started but then held for abscess drainage by interventional radiology. Blood cultures eventually returned after 21 hours and were consistent with *E. rhusiopathiae *bacteremia. Repeat blood cultures, taken three days later, showed no growth, and intravenous vancomycin was discontinued as the E. rhusiopathiae was found to be sensitive to ceftriaxone, with a minimum inhibitory concentration of ≤0.25 µg/mL, in line with antimicrobial stewardship. She was then started on a six-week course of intravenous ceftriaxone 2 gm daily.

Further cardiac imaging was obtained due to concern for possible endocarditis. A transthoracic echocardiogram (TTE) revealed a preserved left ventricular ejection fraction, a small to moderate-sized irregular, poorly defined echogenicity on the LV outflow tract side of the valve, a small, mobile vegetation or torn flail chordae on the atrial aspect of the mitral valve, severe mitral stenosis, and a severely dilated left atrium (Video 1). The pacemaker lead was noted in the right ventricle with a small mobile echogenicity suggestive of vegetation. However, this was later reevaluated and ruled not to be vegetation. The aortic valve was found to have a mean systolic gradient of 10 mmHg and the mitral valve was found to have a mean diastolic gradient of 13 mmHg. The infectious disease team had initially recommended a transesophageal echocardiogram (TEE) for further evaluation of the mitral valve, but cardiology said that a TEE would not likely change the clinical management.

**Video 1:** TTE in apical 4-chamber view: demonstrating small, mobile vegetation or torn flail chordae on the mitral valve, severe mitral stenosis (on the right side of the video), and a severely dilated left atrium (in the bottom right chamber) TTE: transthoracic echocardiogram

The source of the bacteria was assumed to be an open wound on her hand, which could have been contaminated while gardening. After discussion with the infectious disease team, they did not think it likely that there was any other possible source due to the bacteria's known vectors of the bacteria. Given these findings, electrophysiology recommended open heart surgery to remove the infected bioprosthetic aortic valve and mitral valve replacement as well as removing the Micra. The patient was deemed too high of a risk for cardiac surgery due to her age, functional status, and comorbidities, and the patient was instead discharged to a skilled rehabilitation facility. She was started on Eliquis for her new-onset atrial fibrillation. The severity of the patient's penicillin allergy was unspecified in the chart, but when asked the patient did not recall a previous anaphylactic reaction so the infectious disease team felt comfortable prescribing a third-generation cephalosporin and she was sent home with a six-week course of IV ceftriaxone. 

She was readmitted to the hospital after three months for a congestive heart failure (CHF) exacerbation. At that time, her infection had resolved. 

## Discussion

Over 200 *E. Rhusiopathiae* human infections have been reported worldwide [4]. A systematic review of case reports from January 2000 to November 2020 identified 62 unique patients with *E. rhusiopathiae* infection, of which 18 cases noted endocarditis or heart valve regurgitation. Of those 18 cases, only nine underwent aortic valve surgical correction. There were five patients who underwent mitral valve surgery (one patient underwent mitral and aortic valve surgery) and one patient who had tricuspid valve surgery. All patients who underwent surgical valve replacement recovered, except for the one who had tricuspid valve surgery and died. The only other patient who died of the 62 unique cases, who was found to have cardiac valve involvement, did not undergo surgery and was treated medically [5]. In a Pubmed search of case reports, only one patient had a noted history of aortic valve repair; however, there was no evidence of endocarditis on the echocardiogram, suggesting a lack of involvement [6]. Additionally, in a literature review, two cases highlight patients who had both endocarditis and osteomyelitis [7,8]. The patient's case is notable for its complexity and highlights challenges in managing *E. rhusiopathiae* infections.

Standard management of infected implantable devices and bioprostheses usually includes removal of the device, surgical washout, and prolonged antibiotic treatment. In this case, given the patient's significant thoracic surgical history (CABG, TAVI, and pacemaker) and baseline functional status, including her recent neurological decline, she was a poor surgical candidate. Had surgical intervention been an option, the removal of the Micra pacemaker and valve replacement would have been a complicated set of procedures. Additionally given her other comorbid conditions with poor prognosis, the question arose on whether surgical exchange of TAVI and pacemaker would even be an ethical option. When reviewing the literature, surgery was deemed necessary in the majority of cases of *E. rhusiopathiae* endocarditis (57% of cases), and mortality was high (33%) [3]. As procedures to install implantable devices become more easily tolerated, and low-risk procedures are done with increasing frequency, it would be wise to assume that bacterial invasion of these devices in the setting of bacteremia will increase in frequency as well. A more established protocol for rare bacteria may be necessary. Clinicians must also consider the ethical principles of beneficence and non-maleficence in the context of their patient's presentation before proceeding down either a medical or surgical approach to management. Although literature points to successful surgical outcomes, any time a patient is brought to surgery they are put at risk of complications related to anesthesia, bleeding, infection, and procedure-specific complications among other risks. These conversations will necessitate a multidisciplinary team approach involving cardiology, infectious disease, surgery, medicine, and other specialties such as palliative care. This is one of very few published cases of *E. rhusiopathiae* with co-occurring osteomyelitis. This raises the question of the order of bacterial seeding and hematogenous spread, as well as the effectiveness of device removal when secondary seeding is present at other locations. It is most likely that the patient became bacteremic and, through hematogenous spread, developed endocarditis and osteomyelitis. Several studies have shown that musculoskeletal complaints can often be the presenting symptom in patients found to have concurrent endocarditis and osteomyelitis [9,10]. However, further research can help elucidate this point. It is important for clinicians to be more aware of* E. rhusiopathiae *as a pathogen, especially in populations at highest risk such as those who garden, those who handle animals, and those living in agricultural communities. 

## Conclusions

This case of an 86-year-old female with a complex *E. rhusiopathiae* infection highlights several critical aspects of managing invasive bacterial diseases, particularly in the presence of prosthetic devices and severe comorbid conditions including both clinical and ethical considerations. This patient's multifaceted presentation, including bacteremia, bioprosthetic aortic valve endocarditis, native mitral valve endocarditis, and lumbar osteomyelitis/discitis, underlines the pathogen's potential for widespread dissemination and the challenges associated with its treatment. Her case is particularly notable due to the involvement of a TAVI, marking a rare instance in the literature where implantable cardiac devices are affected by this bacterium. The successful resolution of the patient’s infection via surveillance blood cultures and repeat TTE following a six-week course of IV ceftriaxone, despite her inability to undergo surgical intervention due to her high surgical risk and complex medical history, provides valuable insights into the non-surgical management of endocarditis associated with *E. rhusiopathiae*. It emphasizes the potential of conservative management with prolonged antibiotic therapy in cases where surgery poses too great a risk to the patient. This case's addition to the literature can help guide future decisions in patients with similar presentations. Furthermore, this case serves as a reminder of the critical need for vigilance regarding traditionally zoonotic infections, especially in elderly patients or those with significant exposure histories, and underscores the importance of considering such pathogens in atypical presentations of device-related infections. 

Several research questions and guideline gaps were identified (optimal antibiotic regimens for prosthetic device-related infections or the role of imaging in monitoring response). Should histories of zoonotic exposure, gardening, or other high-risk exposures be routinely screened for in patients undergoing assessment for cardiac implants? Developing guidelines for determining when to pursue surgical intervention versus conservative management in patients with endocarditis and cardiac implants, particularly for rare bacterial cases, may be essential for optimizing patient care. Further research is needed to address these gaps and inform evidence-based guidelines that improve patient outcomes and standardize care in these complex clinical scenarios. In summary, this case not only adds to the sparse data on *E. rhusiopathiae* infections involving endocarditis and osteomyelitis but also prompts reconsideration of management strategies in the aging population with multiple comorbidities and implanted devices. The insights gained here should encourage further research and discussion on optimizing care for similar complex cases, potentially influencing future guidelines on the management of rare bacterial infections in a setting increasingly dominated by advanced medical and surgical interventions.
